# Computed tomography micromotion analysis in the follow-up of patients with surgically treated pelvic fractures: a prospective clinical study

**DOI:** 10.1007/s00590-023-03542-w

**Published:** 2023-04-14

**Authors:** Natalie Lundin, Henrik Olivecrona, Peyman Bakhshayesh, Lena Gordon Murkes, Anders Enocson

**Affiliations:** 1 Department of Molecular Medicine and Surgery, Karolinska Institute, Stockholm, Sweden; 2 Department of Trauma, Acute Surgery and Orthopedics, Karolinska University Hospital, Stockholm, Sweden; 3 Leeds General Infirmary Major Trauma Centre, University of Leeds, Leeds, UK; 4 Department of Pediatric Radiology, Astrid Lindgren Children’s Hospital, Karolinska University Hospital, Stockholm, Sweden

**Keywords:** Pelvic fracture, Surgical treatment, Trauma, Low-dose CT

## Abstract

**Purpose:**

High-energy pelvic fractures are complex injuries often requiring surgical treatment. Different radiological methods exist to evaluate the reduction and healing process postoperatively but with certain limitations. The aim of this study was to evaluate Computed Tomography Micromotion Analysis (CTMA) in a clinical setting for follow-up of surgically treated pelvic fracture patients.

**Methods:**

10 patients surgically treated for a pelvic fracture were included and prospectively followed with Computed Tomography (CT) at 0, 6, 12 and 52 weeks postoperatively. CTMA was used to measure postoperative translation and rotation of the pelvic fracture during the 52 weeks follow-up. Clinical outcomes were collected through the questionnaires EQ-5D index score and Majeed score.

**Results:**

10 patients were included with mean age (± SD, min–max) 52 (16, 31–80) years and 70% (*n* = 7) were males. The median (IQR, min–max) global translation from 0 to 52 weeks was 6.0 (4.6, 1.4–12.6) millimeters and median global rotation was 2.6 (2.4, 0.7–4.7) degrees. The general trend was a larger translation between 0 and 6 weeks postoperatively compared to 6–12 and 12–52 weeks. For the clinical outcomes, the general trend was that all patients started from high scores which decreased in the first postoperative follow-up and recovered to different extent during the study period.

**Conclusion:**

CTMA was successfully used in the follow-up of surgically treated pelvic fracture patients. Movement in the pelvic fractures after surgical fixation was largest between 0 and 6 weeks.

## Introduction

High-energy pelvic fractures are complex injuries often causing long-term pain and disability in the affected individual [1]. Surgical treatment for these patients aims at restoring the anatomy and stability of the pelvic ring and to facilitate mobilization during the healing process [2]. Different radiological methods exist to evaluate reduction and healing of pelvic fractures postoperatively. Challenges in this process are, due to the complexity of the pelvic anatomy, for example evaluating the sacrum and the sacroiliac (SI) joints, as well as finding incongruency in the acetabular region, especially after insertion of osteosynthesis material [3, 4]. Assessment of the pelvis with traditional plain radiography with anteroposterior, inlet and outlet projections have known limitations with poor reliability and accuracy in detecting displacement but are still often used during follow-up of these patients [3, 5]. Computed Tomography (CT) is evidently a superior radiological method compared to plain radiography when evaluating the pelvis and its use is increasing, also in the follow-up, as the radiation doses have been gradually reduced with improved low-dose techniques [4].

The comparatively new but still well-tested method Computed Tomography Micromotion Analysis (CTMA) is a post-processing tool that calculates the difference in translation and rotation between two different CT-stacks using volume registration, hence can distinguish small displacements in millimeters and degrees. The method has so far, like the Radiostereometric Analysis (RSA), mainly been used to detect implant motion in hip arthroplasty. In contrast to RSA, CTMA does not need marker bead implantation and the method uses a true three-dimensional (3D) input data instead of plain radiographs as in classic RSA [6–9]. Previous studies using CTMA have shown a precision in the same order of magnitude as in RSA [7, 9–11]. Current literature on the use of CTMA in the follow-up of fracture patients is as of today non-existing.

In this study, we used CTMA for the first time in a clinical setting in patients surgically treated for pelvic fractures. The aim was to test the feasibility of the method in a clinical prospective study.

## Methods

In this prospective clinical study, we included 10 adult patients with pelvic fracture requiring surgical treatment. All patients were included after informed personal consent and underwent surgical treatment at a University Hospital Clinic from January 2019 to May 2020. Inclusion criteria were age ≥ 18 years and a pelvic ring injury requiring surgical treatment with fixation at two different sites of the pelvic ring. Exclusion criteria were non-residents or any condition that made the patient unable to assess the study information or to take informed personal consent. Collected variables were gender, age, fracture type, surgical fixation method, translation (mm) and rotation (deg) over time and self-reported scores: EuroQol-5D (EQ-5D) index score [12] and Majeed score [13]. Pelvic fractures were classified according to the Young Burgess classification [14].

All patients underwent standard protocol postoperative CT scan and then low-dose CT scan at 6, 12 and 52 weeks postoperatively. Patients were requested to answer questionnaires for EQ-5D index score and Majeed score, first upon inclusion in the study (recall value before injury) and then during follow up visits at 6, 12 and 52 weeks. The CT files were anonymously transferred to a research computer where they were analyzed with the CTMA software.

The first postoperative scan was performed on a GE Revolution CT-scanner. The protocols used varied depending on patient status. Most frequently a dual energy protocol was used with tube voltages switching between 140/80 kV. Additional parameters used were; a pitch of 1.37, 1.0 s rotation time, 80 mm collimation with a fixed tube current resulting in a Computed Tomography Dose Index (CTDIvol) of 8.84 mGy. The follow-up scans were performed on a third generation dual-source CT-scanner (SOMATOM Force, Siemens Healthineers, Forchheim, Germany). A dedicated low-dose protocol was set up. The following protocol parameters were selected: 100 kV with tin filtration, 57.6 mm collimation, a single x-ray source with a pitch of 1.2, 0.5 s rotation time and automated tube current modulation. The organ characteristic was set to thorax with a quality reference mAs of 200 (the vendor’s image quality reference parameter). The effective dose for each patient from the low-dose scan was estimated using a commercial radiation dose estimation software package (ImpactDose, CT Imaging GmbH, Erlangen, Germany), which uses pre-calculated conversion factors based on Monte Carlo simulation.

**Fig. 1:**
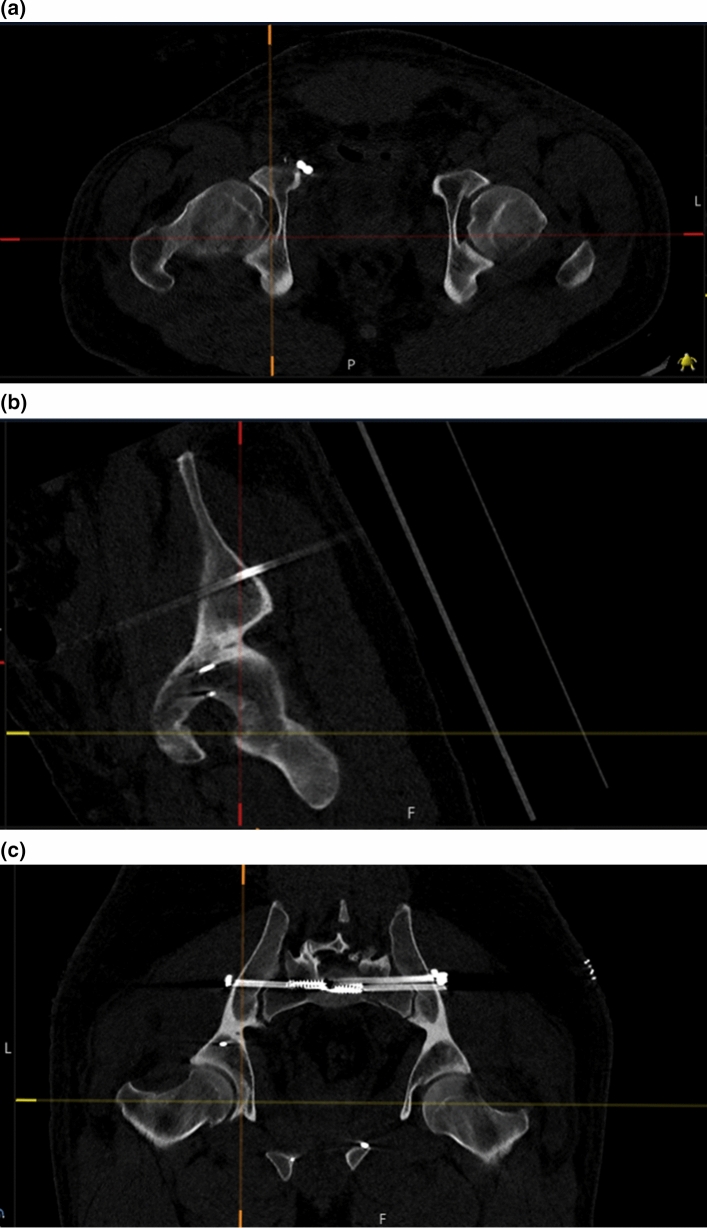
**a** Graphic display of the analysis method and the chosen measurement point in the axial plane. **b** Graphic display of the analysis method and the chosen measurement point in the sagittal plane. **c** Graphic display of the analysis method and the chosen measurement point in the coronal plane

We used the image post-processing tool CTMA (Sectra AB, Linköping, Sweden) to compare differences in the relative position of a designated area in the two hemi pelvises in CT scans comparing 0–6, 6–12, 12–52 and 0–52 weeks. Using the CTMA tool, one “stationary” and one “moving” surface area were chosen to calculate the difference in translation and rotation between these bony regions in the two CT stacks. The system uses volume registration of the selected regions and automated computation of the relative movement between these two rigid bodies. In this study we chose a region around the acetabulum of the least injured side as stationary, and one around the contralateral acetabulum as moving. For the translation analysis, we chose a point in the inferior and posterior part of the contralateral fovea centralis. This point was situated in the quadrilateral plate and was chosen as this area was well aligned in all patients. No patient had an acetabular fracture. We hypothesized that in a stable non-fractured pelvis the translation between these points, and the difference in orientation of the region of the pelvis around these points would be close to zero [8]. Non-zero numbers, higher than expected measurement error, reflect true movement, indicating deformation of the fracture system and thereby a change in the geometry of the pelvic ring over time. The CTMA numerical output gives translation and rotation along three arbitrarily chosen orthogonal axes (X, Y and Z), and as a global translation in chosen point/points as well as global rotation of the segment. In this study the X axis was defined as from left to right, the Y axis from front to back and the Z axis from feet to head as given by the native CT coordinate system. Besides numerical output, the relative displacement can be studied in 2D and 3D images. The outline of the analysis is graphically displayed in Fig. 1a–c. The expected precision of the analysis was graphically evaluated using feedback features provided by the system.

The EQ-5D index score questionnaire was used to assess health-related quality of life and follow-up results were compared to the patient’s preoperative status. EQ-5D uses five clinical dimensions and the values are translated to an overall index score of 0–1, where 1 is the best possible value. We also used the more pelvic injury specific questionnaire, the Majeed score, to assess the clinical outcome of the study patients. In the Majeed score, five factors are assessed; pain, standing, sitting, sexual intercourse and work performance, and the score ranges from 0 to 100 were 100 is the best score.

### Statistical analysis

Numerical data were presented as mean (± SD, min–max) or median (IQR, range). We refrained from doing statistical testing on the data due to the low number of patients. The statistical software used was Microsoft Excel for Mac, version 16.16.2 and IBM SPSS Statistics, Version 25 for Windows (SPSS Inc., Chicago, Illinois).

## Results

All 10 patients attended all follow-up visits including low-dose CT scans. The mean age (± SD, min–max) was 52 (16, 31–80) years and 70% (*n* = 7) were males. Patient characteristics, fracture classification and treatment are displayed in Table 1.

**Table 1:** Patient characteristics, fracture types and surgical methods of all 10 patients

Patient	Age at inclusion	Gender	Fracture classification	Type of surgical treatment
1	61	Male	LC3	Right pelvic plate bridging the symphysis and bilateral SI-screws
2	32	Male	LC2	Symphysis plate and right os ilium plate
3	80	Female	APC2	Symphysis plate and right SI-screw
4	64	Male	APC2	Symphysis plate, bilateral SI-screw, os ilium screw
5	64	Female	APC2	Symphysis plate and right SI-screw
6	55	Female	LC2	Right pelvic plate and right SI-screw
7	33	Male	APC3	Symphysis plate and right SI-screw
8	48	Male	APC2	Symphysis plate and right SI-screw
9	31	Male	APC2	Right pelvic plate bridging the symphysis and bilateral SI-screws
10	51	Male	APC3	Symphysis plate and bilateral SI-screws

*LC* lateral compression, *APC* anteroposterior compression, *SI* sacroiliac

**Fig. 2:**
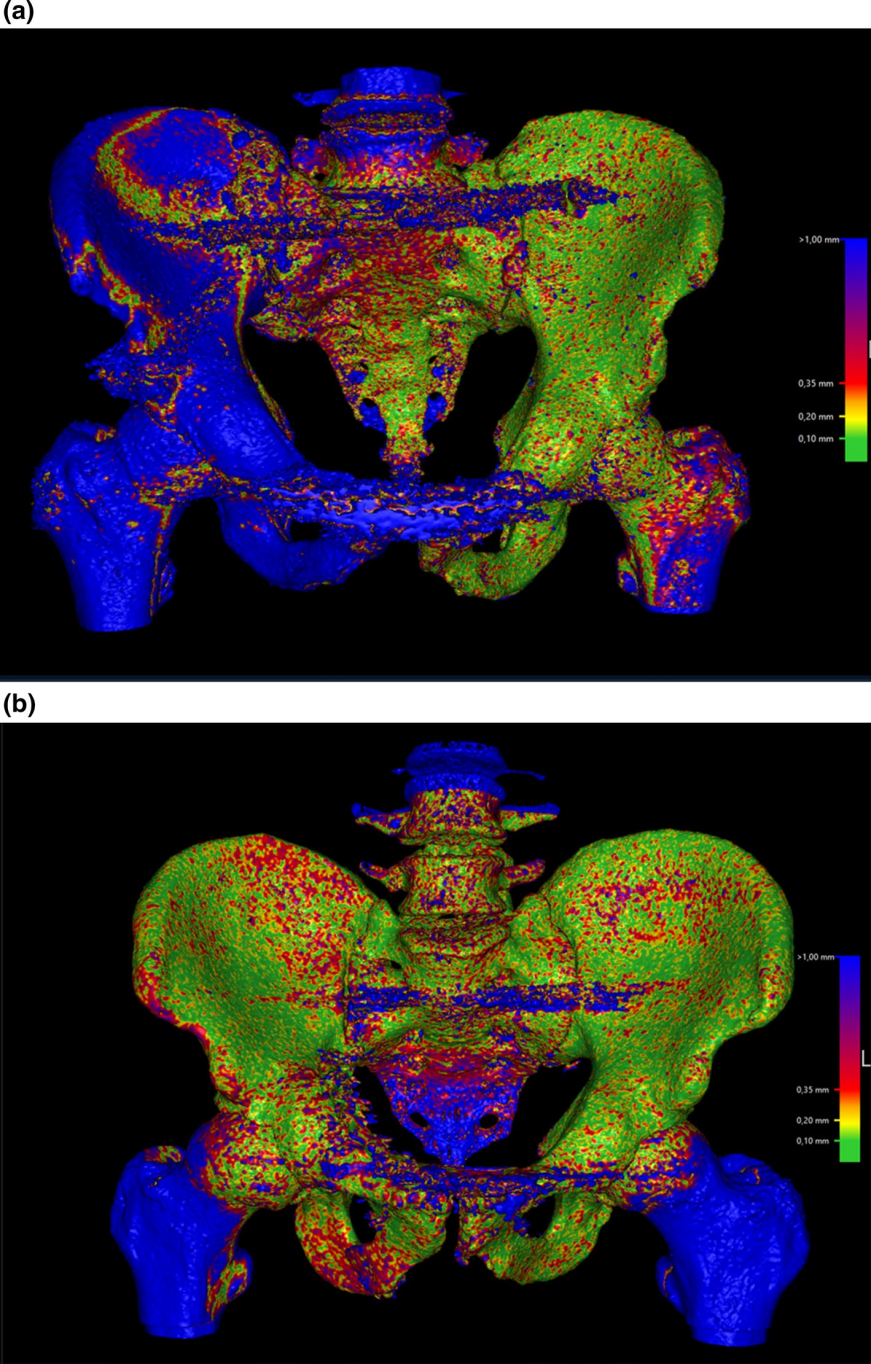
**a** Alignment of patient number 4 between 6 and 12 weeks, illustrating a movement of > 1 mm in the right injured area of the pelvis (blue colored), compared to the left uninjured side. **b** Alignment of patient number 9 between 6 and 12 weeks, illustrating a movement of < 0.2 mm in the right injured area of the pelvis (green colored), compared to the left uninjured side (colour figure online)

The median (IQR, range) translational (mm) and rotational (deg) changes in the X, Y and Z axes of the 10 patients for the different time intervals are presented in Table 2. The median global translation from 0 to 6 weeks was 4.4 (4.3, 1.6–10.6) mm and median global rotation was 1.7 (1.6, 1.1–4.2) deg (Table 2). The median global translation from 6 to 12 weeks was 1.9 (2.7, 0.2–4.3) mm and median global rotation was 1.1 (1.4, 0.2–2.0) deg (Table 2). The median global translation from 12 to 52 weeks was 1.6 (3.9, 0.6–5.6) mm and median global rotation was 1.6 (1.0, 0.5–2.8) deg (Table 2). The median global translation from 0 to 52 weeks was 6.0 (4.6, 1.4–12.6) mm and median global rotation was 2.6 (2.4, 0.7–4.7) deg (Table 2). The detailed translational and rotational changes in the X, Y and Z axes for all 10 patients individually during the entire period, 0–52 weeks, are presented in Table 3. The general trend was a larger translation between 0 and 6 weeks postoperatively compared to 6–12 and 12–52 weeks (Table 2), and larger translation values along the Z axis (Table 2). A similar trend was noted for the global rotation, with largest rotation between 0 and 6 weeks (Table 2). Individual differences in translation and rotation were noticeable with translation of between 1.4 and 12.6 mm and rotation of between 0.7 and 4.7 deg from 0 to 52 weeks (Table 3). Figure 2a, b provides visual example of two patients illustrating both a larger and a smaller motion of the injured right hemi pelvis.

**Table 2:** Median (IQR), Min–Max translation and rotation of the injured hemipelvis of all 10 patients for the different time intervals

Time period	Translation X (mm)	Translation Y (mm)	Translation Z (mm)	Translation global (mm)	Rotation X (deg)	Rotation Y(deg)	Rotation Z (deg)	Rotation global (deg)
0–6 weeks	0.6 (3.9), − 5.3–3.5	0.1 (2.4), − 2.7–6.0	2.5 (3.3), − 1.0–9.2	**4.4 (4.3), 1.6–10.6**	0.1 (1.4), − 1.5–1.1	1.0 (2.2), 0.2–3.4	− 0.4 (2.6), − 2.8–1.9	**1.7 (1.6), 1.1–4.2**
6–12 weeks	− 0.0 (1.7), − 1.9–2.5	− 0.75 (2.0), − 3.3–0.4	0.2 (1.7), − 0.5–2.7	**1.9 (2.7), 0.2–4.3**	− 0.3 (1.2), − 1.6–0.3	0.1 (1.1), − 0.6–1.4	− 0.0 (0.7), − 0.4–1.6	**1.1 (1.4), 0.2–2.0**
12–52 weeks	0.2 (1.4), − 2.3–3.6	− 0.1 (1.4), 3.7–0.6	0.1 (3.0), − 1.1–5.4	**1.6 (3.9), 0.6–5.6**	− 0.2(1.1), − .4–1.2	− 0.4 (2.3), − 2.1–2.5	− 0.0 (1.2), − 0.6–1.2	**1.6 (1.0), 0.5–2.8**
0–52 weeks	0.4 (5.9), − 3.5–6.1	− 1.1 (5.2), − 6.7–5.8	3.4 (2.9), 0.1–10.1	**6.0 (4.6), 1.4–12.6**	− 1.3 (1.7), − 2.2–2.1	0.7 (2.7), -1.6–2.4	0.0 (3.6), − 1.5–4.1	**2.6 (2.4), 0.7–4.7**

Bold text highlights the median (IQR), Min–Max global translation and rotation

*IQR* interquartile range, *mm* millimeters, *deg* degrees

**Table 3:** Translation and rotation of the injured hemipelvis between the first and the 52-week postoperative CT for all 10 patients

Patient number	Translation X (mm)	Translation Y (mm)	Translation Z (mm)	Translation global	Rotation X (deg)	Rotation Y (deg)	Rotation Z (deg)	Rotation global
1	6.1	5.8	0.9	**8.5**	2.1	− 0.6	4.1	**4.7**
2	− 0.2	− 0.2	3.1	**3.1**	− 0.2	2.1	0.8	**2.3**
3	− 3.3	− 0.4	3.8	**5.1**	− 1.3	2.4	− 1.5	**3.1**
4	− 3.5	− 6.7	10.1	**12.6**	− 2.2	1.3	− 1.2	**2.9**
5	− 0.7	− 1.3	0.1	**1.4**	− 0.1	0.7	0.0	**0.7**
6	5.6	− 4.4	2.2	**7.5**	− 2.1	− 1.3	4.0	**4.7**
7	− 2.6	− 6.1	3.6	**7.6**	− 1.8	1.8	2.3	**3.5**
8	2.3	− 1.4	1.9	**3.3**	− 1.4	− 1.6	− 0.8	**2.2**
9	1.1	2.0	3.9	**4.5**	− 1.3	0.2	− 0.8	**1.5**
10	1.5	− 0.9	6.6	**6.8**	− 0.8	0.7	0.1	**1.1**
All Median (IQR), Min–Max	**0.4 (5.9), − 3.5–6.1**	**− 1.1 (5.2), − 6.7–5.8**	**3.4 (2.9), 0.1–10.1**	**6.0 (4.6), 1.4–12.6**	− **1.3 (1.7),** − **2.2–2.1**	**0.7 (2.7),** − **1.6–2.4**	**0.0 (3.6),** − **1.5–4.1**	**2.6 (2.4), 0.7–4.7**

Bold text highlights the global translation and rotation for each patient and the median (IQR), Min–Max translation and rotation of all 10 patients

*Mm* millimeters, *deg* degrees, *IQR* interquartile range

EQ-5D index score and Majeed score at 0 (preoperative score), 6, 12 and 52 weeks postoperatively are presented in Table 4. A general trend was that all patients started from high scores which decreased in the first postoperative follow-up and recovered to different extent during the study period (Table 4). All patients with EQ-5D index score below median value at 52 weeks (patient 1, 6, 7 and 10, Table 4) displayed translation above median between 0 and 52 weeks (Table 3). A similar trend was seen for Majeed score, with the patients 1, 7 and 10 reporting values below median at 52 weeks (Table 4). Patient 3 also reported Majeed score below median, 84/100 at 52 weeks, but was not in occupation (age 80 years) and so could never score maximum 100 points (Table 4). Patient 4 was the only exception with global translational values above median but still reported EQ-5D index score and Majeed score above median at 52 weeks (Tables 3 and 4).

**Table 4:** EQ-5D index score and Majeed score for patient number 1–10 preoperatively (0) and at 6, 12 and 52 weeks postoperatively

EQ-5D index score	0 weeks	6 weeks	12 weeks	52 weeks	Majeed score	0 weeks	6 weeks	12 weeks	52 weeks
Patient					Patient				
1	0.89	0.74	0.77	0.71	1	90	47	78	78
2	1.00	0.81	0.85	1.00	2	100	78	93	100
3	1.00	0.88	0.92	0.89	3	84	58	89	84
4	1.00	0.81	0.81	1.00	4	100	43	58	98
5	1.00	0.81	0.85	0.89	5	100	42	64	100
6	1.00	0.66	0.81	0.78	6	100	47	67	98
7	1.00	0.67	0.67	0.81	7	100	39	52	63
8	1.00	0.85	1.00	1.00	8	100	69	98	100
9	1.00	0.83	0.74	0.89	9	100	56	60	95
10	1.00	0.70	0.66	0.74	10	96	56	49	56
All Median (IQR), Min–Max	**1.00 (0), 0.89–1.00**	**0.81 (0.14), 0.22–0.88**	**0.81 (0.14), 0.66–1.00**	**0.89 (0.23), 0.71–1.00**	**All Median (IQR), Min–Max**	**100 (6),84–100**	**52 (18), 39–78**	**66 (34), 49–98**	**97 (26), 56–100**

Bold text highlights the median (IQR), Min–Max scores of all 10 patients

EQ-5D: EuroQol group-5 Dimensions, IQR: interquartile range

**Table 5:** Effective radiation dose (E) and Computed Tomography Dose Index (CTDIvol) for each follow-up

Weeks postoperative	E (mSv)	CTDIvol (mGy)
	Median (IQR), Min–Max	Median (IQR), Min–Max
0	3.3 (0.8), 0.9–21.7	8.8 (0), 4.4–17.3
6	0.5 (0.2), 0.3–0.6	1.4 (0.3), 1.1–1.8
12	0.4 (0.2), 0.3–0.6	1.2 (0.3), 0.9–1.5
52	0.4 (0.1), 0.3–0.5	1.2 (0.1), 1.0–1.9

*mSv* millisievert, *mGy* milligray, *IQR* interquartile range

Median effective radiation dose and Computed Tomography Dose Index (CTDIvol) for the 10 patients for the four different time points (first postoperative, 6 weeks postoperative, 12 weeks postoperative and 52 weeks postoperative) are presented in Table 5. Median effective radiation dose was 0.4 to 0.5 millisievert (mSv) in the low-dose CTs. The first postoperative CT was performed on standard protocol and not low-dose, hence the higher radiation values. One patient performed the postoperative CT of the pelvis together with other simultaneous investigations resulting in markedly higher median effective radiation dose and CTDIvol (Table 5).

## Discussion

In this study we confirmed the usability of CTMA in a clinical setting. We were able to distinguish motion in the injured part of the pelvis over time in patients surgically treated for a pelvic fracture. The largest global translation (median 4.4 mm) was found between 0 and 6 weeks after surgery, compared to the following time periods. Overall translation was largest along the z-axis (from cranial to caudal), which we interpret as being a result from physiological axial loading. A trend toward a larger overall global translation and rotation, and lower final clinical outcome scores was noted, suggesting a potential correlation between larger movement and worse clinical outcome.

Although not extensively described in the literature, the CTMA technique and its clinical application has been previously studied. A predecessor to this study was the study by Bakhshayesh et al. from 2019 investigating the use of CTMA in pelvic cadavers with simulated fractures. That study proposed excellent precision and repeatability in measuring translation and rotation between fixed points in the pelvic girdle. Using this knowledge, we were now able in this current study to use CTMA in the clinical follow-up of pelvic fracture patients. Bakhshayesh et al. also verified the use of CTMA in comparing contralateral tibias and hemi pelvises in patients, were the results indicated symmetry between both sides [7–9].

Additionally, the CTMA method has previously been evaluated in a clinical setting for detecting early migration of hip implants by Brodén et al. [9, 10]. In their clinical study from 2021, 10 patients underwent both CTMA and RSA after total hip arthroplasty (THA) and the findings suggested a comparable precision between the two methods [9].

Due to the size of our study cohort, we must be cautious to draw firm conclusions, still we found a tendency toward a larger translation during the first 6 weeks after surgery, with subsiding magnitude thereafter. This course of movement can possibly be interpreted as a proxy for fracture healing and could support limited weight bearing during this time period. Fracture healing is of today not properly defined but relies on different criteria with the assessment of the mechanical stability as one important factor. Currently, there is no true consensus on when this stability is achieved (a fracture is healed), although different methods exist to evaluate increased stiffness during fracture healing [15, 16]. A complicating factor in this process for a pelvic fracture is the fact that a pelvic injury might not constitute an actual bone fracture but instead a ligamentous injury, which demands other methods to detect healing than just simply callus formation. Our data with larger translation in the first 6 weeks suggest a larger mechanical instability during this period, and that a gradually increased stability was achieved during the healing process. Further studies with larger number of patients are required however to potentially confirm this finding, and possibly find a cut-off value for when a fracture might be considered healed. This could aid in when to allow full weight-bearing for fractures of the lower extremity, such as femur fractures, tibia plateau fractures or tibial diaphyseal fractures, and possibly minimize follow-up radiographs investigating callus formation.

The clinical outcome, measured through EQ-5D index score and Majeed score, displayed lowest scores at the 6-week postoperative follow-up with an increasing trend thereafter up until the final follow-up after 1 year. A few patients deviated from this trend, for example patient number 10 who reported lower scores at the 12-week follow-up and did not recover to the same extent as the majority of the other study participants. A tendency toward a larger overall global translation and lower final clinical scores was noted, with patients reporting lower than median EQ-5D index scores at 52 weeks displaying among the largest translation values during the entire study period. Patient 4 was an exception to this pattern. This study participant displayed the largest overall global translation (12.6 mm) but still reported almost maximum scores at the 1-year follow-up. Clinical outcome is difficult to predict and relies on several factors apart from the healing of the injury, such as pre-injury score, presence of neurological injury, age and gender [17, 18, 18], and does not always correspond to the radiographic outcome [19]. Our clinical outcome scores at 1 year were comparable but in the higher range of existing literature, although without completely comparable cohorts and follow-up times [17, 18, 18]. In our material, no patient underwent any reoperation during the first postoperative year, which is otherwise fairly common among surgically treated pelvic fracture patients [20]. This indicates a potential selection bias in our study population, in favor of an overall less severely injured cohort of patients, possibly explained by the fact that we could not include patients not well enough to take informed personal consent due to other concomitant injuries or conditions. It could also relate to the relatively short follow-up time (one year). As a next step, a larger more unselected cohort of surgically treated pelvic fracture patients would be needed to follow with CTMA, and for a longer time period, to further investigate a potential relationship between movement and clinical outcome. In this way we might be able to establish an association and to find cut-off values related to worse outcomes, such as risk for reoperations.

In our material, all patients had one intact unfractured hemipelvis, and the uninjured acetabulum of that side was chosen as the reference area in the CTMA analysis. Bilateral injury of the pelvis was not an exclusion criteria, but would have rendered the use of another reference area, such as possibly the sacrum.

The calculated median effective radiation dose for the low-dose protocol in our study (0.4–0.5 mSv) was in the lower range compared to other studies using low-dose CT of the pelvis (0.2–2.3 mSv) [4, 9, 10]. Our values can be compared to effective radiation doses of between 0.3 and 0.4 mSv for CR of the pelvis with 3–5 projections in recently reported data [4, 23]. Attaining correct and comparable projections of the pelvis with CR can be challenging, and incorrect projections might demand repeated radiography in 6–20% of cases, resulting in higher radiation doses and patient inconvenience [21]. It is of course important to critically evaluate every radiography performed, either CR or CT, to properly define the clinical use and balance the risk of radiation versus patient benefit. The Swedish Radiation Safety Authority, working to ensure radiation safety in Sweden, reports continuously decreasing radiation doses from the beginning of the twentieth century up until 2019, for both CR (30–50%) and CT (25–40%), partly attributed to the technical development and improvement of image detectors [22]. Our radiation data with low-dose CT values at similar levels as CR with several projections of the pelvis, together with anticipated continued improvements in low-dose-CT technique, confirm the probable increase in use of CT as the modality of choice for pelvic fractures in future. CTMA is then likely to be a standard tool in the follow-up of both pelvic and other complex fractures.

It could be debated whether pelvic fracture patients benefit from radiological follow-up at all and to what extent after the first postoperative radiography, with the small but still present radiation dose present with every extra examination, CR or CT. It could be argued that only patients deviating from standard clinical outcome should undergo further radiography after the first postoperative follow-up. Still, pelvic fracture patients belong to a heterogonous group of patients, often severely injured with not only their pelvic fracture, and at high risk of postoperative complications. We believe that they need best possible follow-up, both clinically and radiologically to early detect implant loosening/osteosynthesis failure or healing disturbances, as delayed diagnosis of these conditions could interrupt the already long rehabilitation period for these patients. Additionally, it is important that all radiographic examination performed provide accurate and useful information to the clinician. CTMA using low-dose CT provides a safe method for this, but the number and time frames for follow-up could be elaborated further.

### Strengths and limitations

The major strength of this study was the prospective study design, with which we were able to evaluate CTMA in a clinical setting in pelvic fracture patients for the first time. All study patients were included and followed during the study period by one of the two authors (AE and NL) and all low-dose CT examinations were reviewed by the author LGM, ensuring consistency in the data collecting process. The main limitation was the low number of patients, making it difficult to draw conclusions on associations, but this study was mainly designed as a first feasibility study to investigate the applicability of the method.

## Conclusions

CTMA provides a feasible and functional method to quantify translation and rotation over time for follow-up of patients with pelvic fracture and it might thus be a helpful tool to assess healing. Movement in the injured part of the pelvis after surgical fixation was largest between 0 and 6 weeks.

## Data Availability

Not applicable.
